# The power of informal cancer caregivers’ writings: results from a thematic and narrative analysis

**DOI:** 10.1007/s00520-020-05901-3

**Published:** 2021-01-09

**Authors:** Nicoletta Suter, Giulia Ardizzone, Guido Giarelli, Lucia Cadorin, Nicolas Gruarin, Chiara Cipolat Mis, Nancy Michilin, Alessandra Merighi, Ivana Truccolo

**Affiliations:** 1 Continuing Education Office, Centro di Riferimento Oncologico di Aviano (CRO) IRCCS, I-33081 Aviano, PN Italy; 2 University “Magna Graecia”, Catanzaro, Italy; 3 Scientific & Patients’ Library, Centro di Riferimento Oncologico di Aviano (CRO) IRCCS, Aviano, Italy; 4 “Flora” High School, Pordenone, Italy

**Keywords:** Informal caregiver, Neoplasms, Narrative analysis, Thematic analysis, Storytelling, Expressive writing, Patient engagement, Narrative medicine

## Abstract

**Background:**

Cancer is a disease that disrupts not only the patient’s life, but that of the entire family as well, from a care, organizational, and emotional perspective. Patients share their experience of illness frequently with their informal caregiver (IC), a partner, son/daughter, friend, volunteer, or any other person in the family or social network who offers to support them during their clinical journey. The purpose of this study was to investigate ICs’ still unknown cancer experiences through the stories of IC participants in a Literary Artistic Competition the Centro di Riferimento Oncologico di Aviano (CRO) IRCSS organized, and understand the themes that emerged from their texts and hence, the power of expressive writing.

**Materials and methods:**

A qualitative study was carried out on literary texts using Mishler’s three levels of narrative analysis: thematic (to detect themes and subthemes); structural (to support the thematic level), and performative (to understand the narratives’ meaning). In addition, the narratives were classified based on Kleinman and Frank’s models. A particular focus was placed on the language of the narratives to identify figures of speech, e.g., metaphors related to cancer.

**Results:**

Seven main themes emerged from the 40 stories’ thematic analysis: perceptions of the disease; biographical breakdown; relationships; transformation of the sick body; IC’s role; encounter with death; and strength of memory. The ICs’ stories also highlighted the strengths and weaknesses of the patient’s clinical pathway. ICs are a resource not only for the patient, who, thanks to them, is assured of continuous assistance but also for the healthcare organization, above all because they serve a relational role as a “bridge” between patients and healthcare workers. ICs have important messages to offer to healthcare organizations. If involved adequately, they can provide a strategic strength in supporting patients and healthcare workers themselves. The in-depth analysis of the themes and subthemes in this study led the authors to hypothesize that expressive writing benefit ICs with respect to the possibility of sharing their experiences with others and giving evidence of their role. Their stories are a testimony that can help those who face a similar experience.

**Supplementary Information:**

The online version contains supplementary material available at 10.1007/s00520-020-05901-3.

## Background

Cancer is a disease that disrupts not only the patient’s life, but that of the entire family system as well from a care, organizational, and emotional perspective. Cancer patients share their experiences of illness frequently with their informal caregivers (ICs), but there is no unique and formal definition of who these are. According to PubMed/Medline, e.g., caregivers are both “persons who provide care to those who need supervision or assistance in illness or disability” and health personnel [1]. However, ICs’ importance and challenge to healthcare organizations are recognized increasingly. The target population of our study is ICs who cope with cancer issues and participated spontaneously in a particular type of expressive writing activity.

Cancer ICs are “…any relatives (adult children, parents, siblings), friends, or partners (spouses) who have a meaningful relationship with, and provide physical, psychological, or emotional assistance to, cancer patients over a long period” [2].

There is a growing literature, particularly in the past 25 years, on ICs associated with chronic disease patients [3]. Very often, these ICs are women who fail to take care of their own needs because of the time and logistics required not only to provide physical care for loved ones, but to care for their emotional burdens [2]. From a conceptual perspective, these caregivers have been equated with family (an individual with a blood relationship) [4], but they may also be simply “someone who shares” the patient’s experience of cancer [5].

Some studies have shown that ICs’ social support varies in quality or effectiveness [6], but this may be a coping response that helps ease the emotional and practical burden of the cancer disease experience [7]. However, ICs’ interventions do differ because they have individual differences in skills, motivation, and ability to overcome the difficult situations associated with cancer care [8], and different authors who have considered caregiving a full-time job that imposes a significant burden of responsibility [2, 7, 9, 10] have investigated the high cost of informal care [11] and the need to involve these caregivers in the healthcare team from the moment of diagnosis [5, 12]. Applebaum and Breitbart [2] described the caregivers’ burden as a “…multidimensional biopsychosocial reaction resulting from an imbalance of care demands relative to caregivers’ personal time, social roles, physical and emotional states, financial resources, and formal care resources given the other multiple roles they fulfill” that leads to physical and emotional demands, fear, hopelessness, mood disturbances, anxiety, and in some cases, even depression. Nevertheless, recent studies have suggested that many ICs claim to have experienced strong positive outcomes, such as resilience, personal growth, or re-evaluation of their lives, and found positive meaning in their loved ones’ experiences of suffering [13, 14].

Such self-efficacy, resilience, and optimism have been found to be related directly to reduced psychological distress [13, 15]. Expressive writing has been recognized as a useful practice through which ICs can describe the benefits of their mental and physical health [16], but through a close reading of their written memories, diaries, or postings on social media, several papers have reported the way in which the ICs describe their experience as caregivers or the potential effect of writing as an expression of their caregiving experience or the issues that emerge [17–19]. Moreover, the types of processes in writing activities that promote their wellbeing, as well as their efficacy in reducing stress, are unclear [20] and require further investigation.

The purpose of our study was to investigate the expressive writing of 40 ICs—not only family caregivers—who are coping with cancer experience. At our Institute, the Centro di Riferimento Oncologico of Aviano (CRO) IRCCS, one of the Italian Cancer Comprehensive Centers located in Northeastern Italy, a prompt-based literary “competition” open to patients, healthcare workers, and caregivers has been organized for five editions/years. Its purpose is to encourage expressive creative writing of these different “categories” of people—the participants were free to choose to what category to apply—and learn ways to improve cancer patients’ care from different perspectives through their narrative stories. These goals were explained clearly in the competition’s application form. Given the Centro di Riferimento Oncologico di Aviano (CRO) IRCSS tradition of “honoring” patients’ stories [21], this activity was part of a more complex institutional Program of Patient Education and Empowerment carried out by a Patient Education and Empowerment Group that includes both patients and caregivers [22]. From 2012 to 2017, 208 stories patients, caregivers, and health professionals wrote were gathered, and in 2018, we developed a qualitative study to analyze the stories. Following Kleinman [23], we use the word “illness” primarily, rather than “disease” or “sickness” in this paper to indicate the patients’ and ICs’ experiences with cancer.

The objectives of our study were to improve the understanding of these ICs’ experiences living with cancer patients, gather their perceptions of cancer, and derive some implications for the continuing education of healthcare workers about their roles, expectations, and priorities.

## Materials and methods

The Regional Ethical Committee approved this qualitative study’s protocol in 2018, at which time, we had the participants’ informed consent to use their narrations already. In every edition of the “literary competition,” the organizer provided the potential applicants with certain prompts, as well as specific rules related to the actual goal of the competition (topic, use of metaphors, deadline, selection criteria, the jury panel, award, privacy, and possible use and reuse of their stories). The prompts were only suggestions of ways to write about their experience as a cancer caregiver.

**Fig. 1:**
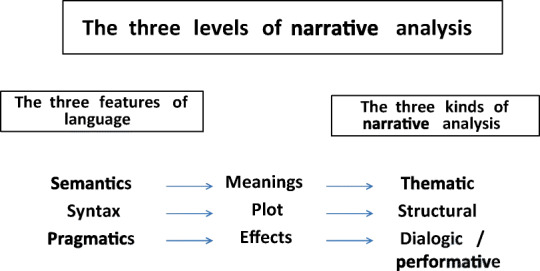
The 3 levels of narrative analysis

First, we chose to begin the analysis with the category of ICs because their perspective is considered in the literature less often than those in other categories. From 2014 to 2017, we gathered 40 stories ICs wrote, all of which were included in this qualitative study: 11 were based on the suggestive prompts “Set sail and travel;” 17 on the prompt “The meeting,” and 12 on the prompt “The names we give to things.” A close reading [24] was performed on the texts, an accurate reading that extends beyond the narrative’s surface to identify the relations between words and the world, meanings, and actions. A research group composed of 9 members with different backgrounds analyzed the stories. A first in-depth reading of the texts took place in subgroups consisting of the group’s supervisor, based on the criterion of integrating different backgrounds to guarantee a variety of findings. The themes that emerged in the subgroups’ analysis were discussed in plenary session, until a final shared interpretation based on the texts was reached. The tool used to update and share methods and information among researchers was a “logbook” that contained the guidelines the members of the research group shared. In addition to monitoring the project, this tool was also used constantly to learn from experience and recalibrate the “emerging design” of the research. Given that language is a social phenomenon, on the basis of the 3 functions of language Halliday [25] described—semantics, syntax, and pragmatics—three levels of narrative analysis were performed following Mishler [26] (Fig. 1):

### Thematic analysis

This focuses on the content and highlights themes (Table 1), as well as the semantic relations between/among them. The themes to be highlighted were selected for each story [27], and the summary of the thematic analysis was represented in a map through a network of relations among the themes. As an example, a table of sentences with their meanings, relative themes, and subthemes is provided (see Table 1 and Table 2).

**Table 1:** Educational material on thematic analysis (Guido Giarelli^3^ coauthor)

Step 1	Select words/phrases from narratives that express meanings
Step 2	Assign meanings to specific themes
Step 3	Identify the semantic relations among the themes
Step 4	Represent these relations with thematic cognitive maps according to the network method

**Table 2:** Example of sentences selected from the stories’ text with their meanings, relative themes, and subthemes

Story code number	Literary competition year	Meaningful words and/or sentences	Subthemes	Themes
IC_9	2014	[..] I’m afraid I will not be able to make it	IC’s Fear	IC’s Pain
		I think it would be better to find someone to help you	Seeking Help	IC’s Role
		That makes me move away from your hairless head, from the sight of disease	IC’s Estrangement from the Sight of the Disease	IC’s Coping Response
		From the arrival of death	Premonition of His/her Own Death	Death

### Structural analysis

This shows the plot’s characteristics, unraveling of situations, characters, point of view, complex action, space and time, direction, resolution, and style (see Table 3).

**Table 3:** Educational material on structural analysis (Guido Giarelli^3^ coauthor)

Narrative elements	Definition
Abstract	Summarizes the sense of the story
Direction	Time, place, characters, and situation
Complicated events	Critical events, action sequences, turning points, problems (plot)
Assessment	Metacomments and the narrator’s feelings (point of view)
Resolution	How the plot of the story ends
End	End of the narrative and return to reality

### Performative analysis

This analyses the various contexts in which the narratives are produced, together with the social, cultural, psychological, and behavioral consequences (e.g., rituals). It is also used to interpret the results and consists of a critical interpretative analysis of the narrative that investigates the implications from a trans-textual and applied perspective, i.e., the transition from text to action (see Table 4).

**Table 4:** Educational material on performative analysis (Guido Giarelli^3^ coauthor)

Step	Definition
Who is speaking?	Who is the narrative’s subject
Who is s/he speaking to?	The narrative’s interlocutor
When is s/he speaking?	The time in the narrator’s life
Why is s/he speaking?	The narrative’s purposes
Where is s/he speaking	The narrative’s context

A list of all of the metaphors that emerged in the various texts analyzed was created (See Table 5, which shows some of these). Finally, an analysis of the ICs’ stories was conducted using Kleinman and Frank’s models [23, 28]. The texts are mentioned according to the year in which they were written and their arrival number.

**Table 5:** Metaphors, figures of speech

Sentences with figurative meaning	Samples
“I was sitting, I think, suspended in the void, floating, in free fall towards the center of the Earth.”	[2015_10_*A chi piacciono i lamponi*.]
“Regressions. Crazy splinters. Glimmers of hope, dazzling flashes of joy. What strength, what wonder, Mom.”	[2015_17_ *Cara mamma*]
“[…] a new friend would keep us company, they introduced her to us shortly before leaving: Sir, this is Chemo, she will travel with you. [...] the undisputed Queen, the silver ampoule, poison in the state pure.”	[2016_9_*I sorrisi permettono all’anima di respirare*]
“I hated that stormy black cloud, loaded with fists of hail as big as tennis balls, sentencing without holds and appeals; I wasn’t ready to face this mountain road so narrow, so steep and so winding.”	
“And the more the years go by, the more you try to fossilize those moments, to recall them, to photograph them and to penetrate the truth (what truth?!) in hindsight, with that lucidity that time imposes on you, with that reasoning that everything explains, to which everything gives its name, that pits everything, demanding answers that never satisfy the soul.”	[2014_5_ *Farfalle*]
“[…] in those four years everything had been chaos and confusion, and attempts to find a way through the rubble [...] his desperate need to save himself meant that he would not let himself be completely swallowed up by that whirlwind of memories.”	[2014_6_*Respiri*]

## Results

Of the 40 IC authors of the stories, 9 were male and 31 female. The relationship between the ICs—narrator—and patient as it emerged from the narration, allowed us to identify the relationship between the subjects: 18 ICs are children of the patients, 8, friends, 7, spouses, 2, mothers, grandchildren, and volunteers, and 1, a sister. The narrative voices, i.e., the “speaker”/narrator, are 11 men and 29 women. Seven main themes emerged from the thematic analysis of the 40 texts (see Table 6). We will present the 7 themes related to the cancer perceptions from the ICs’ point of view shortly.

**Table 6:** Themes and subthemes. The number in the parenthesis after the themes represents the number of times that the theme was present in the data

	Themes	Subthemes
1	ICs’ perception of illness (29)	Silent disease
		Denial of the disease
		The disease as taboo
		The meaning given to the causes of the disease
2	Biographical breakdown (26)	Family coping
		The IC’s suffering at the onset of the disease
		The sense of helplessness, loneliness, and abandonment
3	The ICs’ relationships (18)	The relationship between the IC and patient
		The relationship between the IC and healthcare worker
4	The sick body’s transformation (24)	The IC’s attentive eye
5	The IC’s role in the care journey (26)	Support
		Skills for technical performance in terminal diseases
		The effort of caring
		Ethical issues in coping with terminal disease
6	The encounter with death (14)	Premonition of death
7	The strength of memory (10)	The strength of mourning
		The value of testimony understanding

Here, we provide a list of each theme and its related subthemes that emerged, together with a brief description, as well as several quotations taken from the original stories. The same list, completed with some meaningful quotations for every theme and its subthemes, is available in Table 7, Supplementary Material. While the number in the parenthesis after the theme represents the number of times that the theme was present in the data, the code at the end of the quote (e.g., 2014_6) refers to the identifier of the original story in our digital archive of the stories.

### The illness from the ICs’ point of view (29)

ICs’ experience the illness of the family member as an impotent spectator unprepared for the disease’s onset and manifestations, the representation of which is expressed through significant metaphors, such as the worm (2014_5), a natural disastrous phenomenon (2016_ 1), and the journey (2015_ 17).

A relevant subtheme is cancer disease as a taboo: Patients do not like to talk about it, are ashamed, and talk about it only when the cancer is advanced. In the family, the disease is hidden from the ICs because the patient is afraid of being a “burden” (2014_ 7).

### Biographical breakdown (26)

The diagnosis of cancer causes a radical change in family life, a “tear” between before and after the disease (2016_ 1). After learning of the diagnosis from the doctor or from the family member, many ICs experience physical pain in the stomach and other common symptoms (2016_10). The patient’s experience of illness shocks the ICs, and alternates with fear: their suffering manifests as a sense of helplessness, loneliness, and abandonment, and is mentioned frequently because generally, they hide their feelings from the patient and thus risk increasing their loneliness. They feel overwhelmed by a sense of bewilderment and discouragement because they find themselves without references, and can do nothing to face cancer (2015_ 2). Then, they institute coping strategies derived from their family and cultural background to cope with the patient’s disease by resorting to recreational activities (2015_ 6).

### The sick body’s transformation (24)

During the course of treatment, the IC is a careful observer, a direct witness of the body’s transformation and behaviors, and therefore, of the patient’s person as a whole (2014_ 9, 2015_ 5). A hollow face, gray complexion, and extreme weight loss make the person unrecognizable, which shakes the IC: “[..] I see a long figure covered in white like a ghost [..] it doesn’t seem like my Sara [..]” (2015_ 2; 2015_ 8).

### The IC’s relationships (18)

During the illness, relationships tend to change; some ICs feel the need and importance to have dialogs with their family, and in some cases downplay their role by using irony (2015_ 6). The relationships that are established with healthcare personnel during hospitalizations are fundamental during the course of the treatment. As one said: “You are now at home in [the] ward. Everyone greets you, many remember you. You like this, it makes you feel calm and if you are, I can be calm too” (2016_ 10). However, communication between ICs and doctors is not always effective; the IC tries to have a good dialog with the patient, and pays attention to the use of words: “The big words that doctors say are rocks, towards which I am going to crash [..]” (2014_ 7). The IC often creates a network with volunteer associations to receive support and feel comforted and helped during the most difficult moments (2014_ 1).

### The IC’s role in the care journey (26)

The IC’s role is to care for the patient, accompanying him and also supporting him psychologically during the treatment journey and until his eventual death (2015 _9). In addition, the IC is concerned with cleaning the environment and maintaining confidentiality, and feels reassured if the patient’s dignity is respected (2015_ 15). Within the house, housekeepers, caregivers, and neighbors who tend to the patient’s personal hygiene assist the IC. The IC’s organizational and technical support is fundamental to resolve problems the patient has ambulating and assist him: “[..] had become the updated edition of the instructions to follow for a cancer disease [..] attacking and detaching the oxygen mask, checking the catheters[...]” IV [..] (2015_ 3).

The IC also faces emotionally demanding end-of-life issues, for which s/he does not feel prepared and supported, particularly when decisions important for the family are required. After all, no one is ever ready to approach the death of a loved one, and the IC wonders about the difficult ethical issues with which s/he will have to cope (2016_ 9).

### The IC’s confrontation with the family member’s death (14)

Of the 40 texts analyzed, 24 have their family member’s death in their epilogue, and 7 describe their last moments of life (2014_ 11, 2014_ 1, 2015_ 2). The progression of the disease felt in the patient’s body means resignation for the IC, and although it represents the end of the family member’s suffering, letting go requires a great will and altruism: “I only know that there are, that we are there, if you want to go we are ready [..]” (2014_ 9).

### The strength of memory

Memory helps put the painful events that belong to the past in order: Some ICs ask their deceased loved ones for forgiveness, others reach truths that are difficult to understand, others recall memories of illness experiences that instill trust and courage and allow them to establish a new balance with an exhortative and consoling function. In their death stories, the mourning process takes place through a sort of ritual that only the passage of time permits them: The IC tries to leave behind memories and painful moments and move towards the future for him/herself and the family. Therefore, anecdotes, perfumes, and photos are recalled, and those recollections make their absent family members live eternally and give importance to the value of memory (2014_ 5). This allows ICs to turn to the world with gratitude (2016_ 9).

The analysis of the stories according to Kleinman’s [23] model reveals a prevalence of stories of sickness (in which the focus is on the relational and social point of view) and illness (in which the focus is on the subjective experience). Instead, according to Frank’s [28] model, there is a prevalence of stories of quest (34), i.e., search, discovery. Fewer (5) are stories of restitution, i.e., compensation and reparation, and 1 is of chaos, i.e., disorder, or the so-called antinarratives.

## Discussion

The narratives analyzed are largely life stories that arose from a prompt given to the participants in the different editions of a literary competition/expressive writing initiative as a suggestion to write about their cancer experience as an IC. The results are stories filtered from memory to document and pass on their experience. As Riessman [29] stated, they represent a narrative reconstruction to attribute a meaning to the origin of the disease and to the “biographical rupture” it produces. This reconstruction can take a particular form that is a mixture of facts and fiction, which the reader/researcher’s intervention also affects [30].

The results of the thematic analysis of these narratives confirmed what is described in the literature regarding ICs’ suffering, their role in the cancer patient’s journey, their physical and emotional state while supporting the patient, including at the end of life [31], personal time for caring, and the multiple roles they play [2, 4]. Our stories are filled as well with evidence of resilience [14], personal growth, and reevaluation [13] of ICs’ life [13].

As have other studies [32], our research showed that ICs find themselves unprepared to face important ethical issues related to terminal illness in their loved one’s end of life, but also the ICs’ important role with respect to care benefits. This lack of “skill” is particularly challenging in Italy, given the recent national law about DAT, i.e., the opportunity to decide in advance what kind of treatment a person would like to receive at the end of his/her life [33].

The relationship with healthcare professionals is an aspect of true utility in the patient’s clinical journey. The IC monitors the dosage of the therapy, updates the doctor on the changes in the patient’s body and reactions to treatments, and in case of emergency, supports the doctor when important decisions need to be made.

Further, ICs find themselves giving voice to the patient when the patient is no longer able to communicate his/her needs and desires: The IC is a skilled interpreter of the patient’s nonverbal communication and represents a bridge in patient-healthcare worker communication.

However, the research revealed an important critical issue in communication with the healthcare providers, which in some cases, causes the IC further suffering. Generally, this occurs when the diagnosis is communicated or the IC requests more information on the patient’s clinical status. On the other hand, when the healthcare provider manages to establish good communication with the IC, s/he develops trust in the healthcare personnel and perceives a benefit on an emotional level.

Many studies have indicated that good healthcare is one in which the patient’s point of view is integrated into that of the healthcare provider (i.e., the healthcare worker) and IC. All stakeholders’ engagement in the development of personalized care processes is a way to respect people’s dignity [34]. Our ICs’ stories showed that this integration should be a requirement, not an option for healthcare organizations [27, 35]. Some stories seem to be an actual handbook of good medicine [36] that offers ways to facilitate the integration of the different stakeholders’ perspectives and also honor both the ICs’ role and patients’ stories [22]. However, it is necessary to make room for a type of “narrative humility” [37] on behalf of healthcare workers/decision makers in the healthcare organizations, so that patients’ and ICs’ stories are given the proper place in the construction of good medicine.

## Limitations

This study has many limitations. Informed consent to analyze, use, and reuse the stories was requested and obtained initially from every applicant to the expressive writing competition, in which the competition’s narratives were the source of data. However, we did not collect a great deal of demographic data about the participants or information related to the patients’ precise diagnosis because our principal objective was to encourage people who are experiencing cancer to express themselves while asking them for the minimum data possible. Nonetheless, this is a qualitative study, so that although we did not have a large number of stories, we tried to extract all of the meanings they contained.

## Conclusions

The data that emerged from the textual analysis of the 40 stories written by ICs who participated in the Centro di Riferimento Oncologico di Aviano (CRO) IRCSS literary competition confirmed what has been described with respect to both the physical and emotional burden and the benefits associated with caring for cancer patients. However, certain key messages emerged. The ICs wrote to share their experiences, but also to honor the stories of their sick loved ones and preserve their memories. If included and involved properly, ICs can offer strategic help to healthcare workers that supports patients’ clinical and existential pathway. Furthermore, the ICs often referred in their narratives to their need for support. Often, healthcare workers overlook their burden of suffering and needs, such as the need to have reserved relaxation areas to stay close to their patients, or to be given information about the way to care for their loved ones at home.

Therefore, it will be important to invest in two critical issues this study highlighted: The need to train healthcare workers and provide them with continuing education about terminal care and other cancer issues, as well as to involve ICs adequately in healthcare organizations.

Ultimately, expressive writing appears to be a powerful and inexpensive tool, both to identify ICs’s roles and help healthcare organizations improve themselves.

## Supplementary information

ESM 1 (DOC 49 kb)

## Data Availability

The raw data are the full text stories. Many of them are online available through the Italian database of cancer resources for patients and citizens CIGNOweb.it at the address http://www.cignoweb.it/cro/search?q=antologia+&h=any
