# Supplementary material for: The power of informal cancer caregivers’ writings: results from a thematic and narrative analysis
Source: Support Care Cancer. 2021 Jan 9;29(8):4381–8. doi: 10.1007/s00520-020-05901-3 (PMC8236437; doi:10.1007/s00520-020-05901-3)
Supplement: Supplementary file 1 — (DOC 49 kb) [file 520_2020_5901_MOESM1_ESM.doc]

Table 7. Results

|  | **THEMES** |  |
| --- | --- | --- |
| **1** | THE ILLNESS PERCEIVED BY THE ICs POINT OF VIEW( 29) | ICs experience the illness[[1]](#footnote-2) of the family member as an impotent spectator, unprepared for the outbreak and manifestation of the disease, whose representation is expressed through significant metaphors, such as:  a) the worm: [..] *"corroded by a silent worm that does not forgive, that digs tunnels."* (2014_5);  b) the intruder: *"cursed intruder"* (2015_ 2);  c) a natural disastrous phenomenon: *"[..] disastrous eruption of a volcano believed to be extinct", "earthquake that caused a tsunami"*, (2016_ 1) ";  d) the journey: *"Illness is a journey into the abyss, a putting one's hands in murky waters, where nothing is certain"*, (2015_ 17).  A relevant sub-theme is *cancer disease as a taboo*: patients do not like to talk about it, they are ashamed, they talk about it when the cancer is advanced. In the family, the disease is hidden from the ICs because the patient is afraid of being a "burden":  *"You have cancer. You whispered it to me slowly, as if you were ashamed of it.* "(2014_ 7); *“The tumor [..] My dad introduced me to it. Not that he told me about it, no, for heaven's sake "*(2016_ 3).  *“You waited so as not to bring sadness and pain on my most beautiful day [..] you hid your evil!* [..] (2015_ 17); *"[..] that confession that was held inside [..] without sharing it with anyone [...]"* (2015_ 9).  Another sub-theme *is the meaning that is attributed to the causes of illness*: some beliefs emerge about the cause of the patient's illness, reported by the ICs as the fear of having received punishment from a higher entity, or of having to serve past misconduct, to get a ransom in the present:  *"What kind of punishment was this news? [..]"* (2014_ 1) *"The guilt for making loved ones suffer is so painful as to confuse it with an incurable disease [..]*  (2015_ 15). |
| **2** | BIOGRAPHICAL BREAKAGE | The diagnosis of cancer causes a radical change in family life, a "tear" between before and after the disease:  *"[..] life changed. I was given back a wife in need of everything [..] Eh, yes! Life had changed and not only his: mine too "*(2015_ 9).  *"And even for you, his was only a metastasis, instead it represented a" watershed that separated your life into two parts "*(2016_ 1).  Having learned the diagnosis from the doctor or from the same family member, many ICs experience physical pain in the stomach (in 5 stories the description is similar) and other common symptoms:  *"There is traffic [..] in my thoughts. A coming and going of emotions and fears difficult to manage. The stomach is not late in reminding me that. "* (2016_10).  The patient's experience of illness causes shock in the ICs, fear alternating with anger:  *"The suffering was so great, the fear as well [..]"*, 2015_3)  The suffering of the IC manifests itself as a sense of helplessness, loneliness and abandonment (sometimes within the family itself) and is told frequently because he/she generally hides his/her feelings from the patient thus risking increasing his/her loneliness, he/she feels pervaded from a sense of abandonment, bewilderment, discouragement, because he/she finds himself without references, and can do nothing to face cancer:  *"I felt a sense of impotence inside me, realizing how small and unwary I was [..]"* (2015_ 2);  *"If it had to win [the tumor ed.], it would have won after fighting with all my anger [..], even if I felt powerless"*, 2016_ 9).  *"Storm inside, water of a lake outside",* 2014_ 6)  *"The wife was [..] terribly alone [..] the greatest pain was to face the farewell alone [..]"* (2015_CG_3).  *"Someone could tell me, what to do now? [..] Me and her abandoned [..]. There were no dawns on the horizon. "* (2016_ 9).  Then he/she puts in place coping strategies from his/her family and cultural background to deal with the patient's disease by resorting to literature, poetry, music, aphorisms:  *"If you always look down you will never find rainbows"* (2016_ 2)  *"When I was little, Dad showed me [..] that star so bright that it is Venus. [..] until that light in you will go out the war you will always have won it. In the face of all evil "*(2016_CG_4).  Other strategies concern recreational activities, energy recharge.  *"Distraction and amusements are a cure-all [..] Life en plein air together with many friends and acquaintances had an overwhelming effect on our existence."* (2015_ 6). |
| **3** | THE RELATIONSHIPS OF THE ICs (18) | During the illness, relationships tend to change, some ICs feel the need and importance of a dialogue with their family, in some cases playing down the role through the use of irony:  *"In these moments it is essential to speak, to speak with anyone, about one's own evil, to overcome fears and false modesties [..]"* (2015_ 9)  *"-Eli, just drink! You are so red that you look drunk - [..] A stupid joke, it created relaxation for both of us and we started laughing almost hysterically, but it certainly did us good "*(2015_ 6). The relationships that are established during hospitalizations with healthcare personnel are fundamental during the course of the treatment:  *“You are now at home in ward. Everyone greets you, many remember you. You like this, it makes you feel calm and if you are, I can be calm too. "* (2016_ 10)  However communication is not always effective between ICs and doctors, IC with the patient tries to have a good dialogue, paying attention to the use of words:  *"The big words that doctors say are rocks, towards which I am going to crash [..]"* (2014_ 7.)  *"Instead of chemo I can say therapy, instead of nausea I can say annoyance, instead of anemia I can say slightly low values, instead of weakness I can say slow recovery of strength [..]"* (2016_ 10)  The IC creates a network with associations and bodies in the area that are able to support it, so that it feels comforted and helped in the most difficult moments:  *“I am in contact with the general practitioner, oncologist, cardiologist and pharmacist almost weekly. [..] help me and give me advice "*(2015_ 6).  *"A famous voluntary association [..] communicated to her in real time that they were in possession of a chair [..] it was as if someone had uncovered her skull and lifted her from that weight she could not deal with ... "*(2014_ 1). |
| **4** | THE TRANSFORMATION OF THE SICK BODY(24) | During the course of treatment, the IC is a careful observer, a direct witness of the transformation of the body, of the behaviors and therefore of the patient's person as a whole:  *"You are getting smaller and smaller in the bed that hosts you, you hook me with your eyes"* (2014_ 9).  *"It is excruciating to see how a person transforms himself in such a few days [..]"* (2015_ 5).  A hollow face, a gray complexion, a strong weight loss make the person unrecognizable and the IC is shaken by it:  *"[..] I see a long figure covered in white like a ghost [..] it doesn't seem like my Sara [..]"* (2015_ 2).  *"I had come to see you and, forgive me Valter, I hadn't recognized you"* (2015_ 8). |
| **5** | ROLE OF THE GC IN THE CARE PATH (26 | The role of the IC is to take care of the patient, accompanying him and also supporting him psychologically during the treatment path and / or until death.  *"I found that every step of the way, you took two steps back."* (2016_ 9);  *"If only I could give her help [..] for [..] the indispensable daily push not to abandon herself to apathy"* (2015 _9).  In addition, the IC is concerned with cleaning the environment and maintaining confidentiality, it feels reassured if the dignity of the patient is respected:  *"In the clean and tidy bed, in the privacy of a room [..] in the frequency of visits by nurses [..] Simple, habitual gestures [..] meant that we too entrusted ourselves",* 2015_ 15).  Inside the house the IC is assisted by housekeepers, carers, neighbors for the handling of the personal hygiene of the patient:  *"Walking problems begin, let's call a big man who helps him especially when he has to go to the bathroom."* (2014_ 8).  The organizational and technical support of the IC is fundamental for the resolution of walking problems and patient assistance:  *"[..] had become the updated edition of the instructions to follow for oncological diseases [..] attacking and detaching the oxygen mask, checking the [...] IV [..]* (2015_ 3).  The IC also faces emotionally demanding end-of-life issues, for which he/she does not feel prepared and supported especially when he/she has to make important decisions for its family; after all, you are never ready to approach the death of your family member and the IC wonders about ethical issues that are difficult to deal with:  *"Gag your mouth that could tell you - so no, don't live, let yourself go - I don't have the right. But my heart is screaming."* (2014_ 9).  *"I had to give his warm body a time [..] I made that choice and I never asked myself why"* (2016_ 9). |
|  | THE IC MEETING WITH THE DEATH OF THE FAMILY (14) | Out of 40 analyzed texts, 24 have the death of the family member as their epilogue, 7 describe the last moments of life:  *“Cold [..] hand, long breath. Another, and then a start. Here it is. Everything ended like this ",* (2014_ 11]).  *"The mother turned her eyes towards her, they lit up like two bulbs and turned them forever into the dark"* (2014_ 1).  *"I slowly felt her breathing fleeing from life to advance on the last path that led her to heaven"* (2015_ 2);  The progression of the disease felt in the patient's body means resignation for the IC and although it represents the end of the sufferings of one's family, letting go requires a great will and altruism:  *"I only know that there are, that we are there, if you want to go we are ready [..]"* (2014_ 9). |
| **7.** | THE STRENGTH OF MEMORY(10) | Memory is helpful to put the painful events belonging to the past in order: some ICs ask for forgiveness from deceased patients, others reaches truths that are difficult to understand, others recall memories of illness experiences that transmit trust and courage and lead to re-establishing a new balance with an exhortative and consoling function. In death stories, the mourning process takes place through a sort of ritual that only the passage of time allows to do: the IC tries to leave behind memories and painful moments to move forward the future for him/herself and for the family. Therefore, anecdotes, perfumes, photos are recalled and that recollection makes missing people live eternally and gives importance to the value of memory.  *“I would have liked a thousand surrogates to be able to remember him. Not just pictures. Also voice recordings. Even smells, and those residues that a life leaves behind. "* (2014_ 11).  *"And the more the years go by, the more you try to fossilize those moments, [...] photograph them and penetrate the truth [..] in hindsight, with that lucidity that time imposes on you [..]"* (2014_ 5 ).  ICs turn to the world with gratitude:  *"The affection that has surrounded us is the most vivid memory I have [...] the human solidarity that allows you to find the strength to overcome the greatest adversities"* (2016_ 9).  This is the sentiment of “*pietas*”, which is a feeling of compassion and moved commiseration, the one which human feels towards the sufferings of others who are forced to live with misfortunes. The analysis of the stories according to the Kleinman ^26^ model reveals a prevalence of stories of Sickness (when the focus is on the relational and social point of view of the disease) and Illness (when the focus is on the subjective experience of illness). Instead, according to Frank's model^2^ there is a prevalence of stories of Quest (search, discovery, 34), in a lesser number there are stories of Restitution (compensation, reparation, 5) and of Chaos (disorder, or the so-called anti-narratives, 1). |

1. The authors chose to use mainly the word “illness” (instead of disease) to point out the patients’ and ICs’ experience about cancer, according to A. Kleinman’s “The Illness Narratives: Suffering, Healing, And The Human Condition”, 1989 [↑](#footnote-ref-2)
